# Transplanted Oligodendrocytes and Motoneuron Progenitors Generated from Human Embryonic Stem Cells Promote Locomotor Recovery After Spinal Cord Transection

**DOI:** 10.1002/stem.489

**Published:** 2010-07-27

**Authors:** Slaven Erceg, Mohammad Ronaghi, Marc Oria, Mireia García Roselló, Maria Amparo Pérez Aragó, Maria Gomez Lopez, Ivana Radojevic, Victoria Moreno-Manzano, Francisco-Javier Rodríguez-Jiménez, Shom Shanker Bhattacharya, Juan Cordoba, Miodrag Stojkovic

**Affiliations:** a Cellular Reprogramming Laboratory, Centro de Investigación Príncipe Felipe (CIPF)Valencia, Spain; b CABIMER (Centro Andaluz de Biología Molecular y Medicina Regenerativa), Avda. Americo Vespucio s/n, Parque Científico y Tecnológico CartujaSevilla, Spain; c Liver Unit, Department of Medicine, Hospital Universitari Vall d'Hebron, Universitat Autónoma de BarcelonaBarcelona, Spain; d Centro de Investigación Biomédica en Red de Enfermedades Hepáticas y Digestivas (CIBEREHD), Instituto de Salud Carlos IIIMadrid, Spain; e Spebo MedicalLeskovac, Serbia; f Human Genetics, Medical School University of KragujevacSerbia

**Keywords:** Embryonic stem cells, Oligodendrocytes, Stem cell transplantation, Neural differentiation

## Abstract

Human embryonic stem cells (hESC) hold great promise for the treatment of patients with many neurodegenerative diseases particularly those arising from cell loss or neural dysfunction including spinal cord injury. This study evaluates the therapeutic effects of transplanted hESC-derived oligodendrocyte progenitors (OPC) and/or motoneuron progenitors (MP) on axonal remyelination and functional recovery of adult rats after complete spinal cord transection. OPC and/or MP were grafted into the site of injury in the acute phase. Based on Basso-Beattie-Bresnahan scores recovery of locomotor function was significantly enhanced in rats treated with OPC and/or MP when compared with control animals. When transplanted into the spinal cord immediately after complete transection, OPC and MP survived, migrated, and differentiated into mature oligodendrocytes and neurons showing in vivo electrophysiological activity. Taken together, these results indicate that OPC and MP derived from hESC could be a useful therapeutic strategy to repair injured spinal cord. Stem Cells 2010; 28:1541–1549.

## INTRODUCTION

Spinal cord transection, besides the loss in central control of motor, sensory and autonomic function below the injury site, produces limited exogenous repair and poor functional recovery. The cumulative death of neurons, astroglia, and oligodendroglia in and around the lesion site disrupts neural circuitry and leads to neurological dysfunction [1]. Damaged axons are unable to grow, regenerate and to reconnect themselves with the structures that were innervated before the injury, causing a permanent interruption of the injured nervous route [1–3]. Many factors contribute to this condition including cellular destruction, demyelination, inability to remyelinate spared axons, and failure of axons to overcome the conduction block by astrocyte scar [4–6]. Introducing new cells as progenitors of motoneurons (motoneurons progenitor, MP) or oligodendrocyte progenitor cells (OPC) into a damaged spinal cord to treat a disease could be a good strategy to establish disrupted synaptic connections between the central nervous system (CNS) functions above and below the lesion.

Compared with other stem cell types, human embryonic stem cells (hESC) and induced pluripotency stem (IPS) cells currently show the greatest potential for differentiation and cell replacement therapies. These cells are pluripotent and can give rise to cells of three germinal layers, they can be propagated indefinitely in culture and can provide a large quantity of differentiated cells for transplantation including specific cells of neuronal or glial fates [7–14].

Recently, few studies have demonstrated that transplantation of OPC derived from hESC and MPs derived from mouse embryonic stem cells (mESC) can provide both trophic support for spared axons and participate in remyelination of the injured spinal cord [15–18]. The use of hESC-derived neural progenitors for treatment of spinal cord injury (SCI) has been described [10,19] in which authors showed that transplanted hESC-OPC survived, integrated, differentiated, and remyelated damaged tissue resulting in a significant improvement of locomotor function of rats with spinal cord contusions. However, there is no study that describes transplantation of hESC-OPC and hESC-MP using an animal model with complete spinal cord transection. Therefore, in this study, we sought to evaluate the behavior and efficiency of grafted hESC-OPC and/or hESC-MP into female rats after spinal cord transection.

## MATERIALS AND METHODS

### Cell Culture and Differentiation

Primary hESC colonies (H9, H9-green fluorescent protein (GFP) and H1 lines, WiCell Inc., Madison, WI) were mechanically dispersed into several small clumps, which were cultured on fresh commercially available human foreskin fibroblasts (American Type Culture Collection, Manassas, VA, USA), inactivated by mitomycin C in ES medium containing Knockout-Dulbecco's Modified Eagle Medium (DMEM) (Invitrogen, Carlsbad, CA, http://www.inivitrogen.com), 100 μM ß-mercaptoethanol (Sigma, St. Louis, http://www.sigmaaldrich.com), 1 mM l-glutamine (Invitrogen), 100 mM nonessential amino acids, 20% serum replacement (SR; Invitrogen), 1% penicillin-streptomycin (Invitrogen), and 8 ng/ml basic fibroblast growth factor (bFGF; Invitrogen). Embryonic stem cell (ESC) medium was changed every second day. Human ESC were passaged by incubation in 1 mg/ml collagenase IV (animal-free, Invitrogen) for 5–8 minutes at 37°C or mechanically dissociated and then moved to freshly prepared human foreskin fibroblast layer.

**Figure 1:**
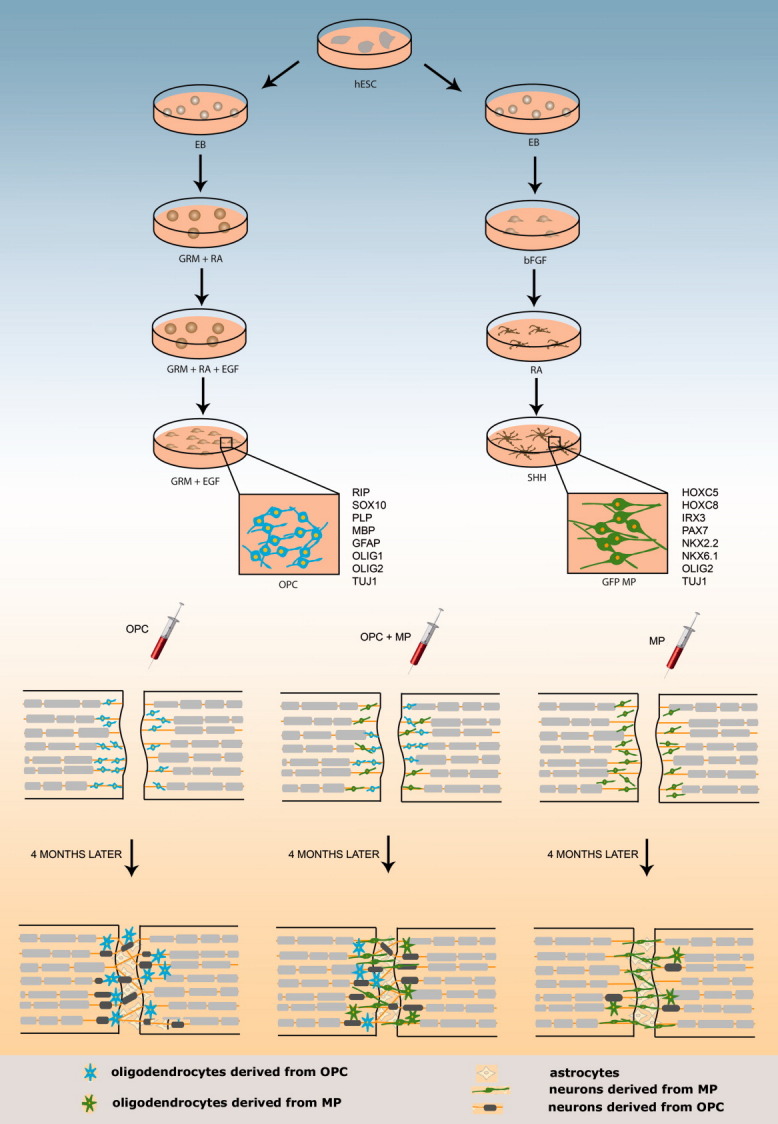
hESCs were differentiated toward OPC in hESC growth media and glial restriction media (GRM) supplemented with EGF and *all-trans*-RA. During 25 days, the EBs were exposed to GRM supplemented with EGF. Floating yellow spheres were plated for 1 week. Cell cultures were replated on Matrigel substrate, and cultured for 1 week in GRM supplemented with EGF. The duration of the protocol was 42 days. For transplantation, the cells were disaggregated mechanically using a glass pipette. For motoneuron differentiation, hESC were permanently transfected with a plasmid carrying GFP. Cell clumps were grown for an additional 4 days to form EBs. The EB were placed on a Matrigel substrate to attach in motor induction medium. From day 8, RA was added. At day 16 the rosettes were placed as round structure-neuromasses in MM consisting of neurobasal medium, N2 supplement, cyclic AMP (cAMP), RA, and SHH. The neuromasses were maintained in MM medium supplemented with SHH until the day of transplantation. Differentiated cells were characterized and ∼1.5 million cells were transplanted. Three different transplantation experiments were performed: The rats that were treated with a single-cell type (OPC or MP) or in combination (OPC + MP). Immunohistochemical evidence of cell incorporation in the LS, and electrophysiological and behavioral studies were performed after 4 months. Abbreviations: bFGF, basic fibroblast growth factor; EB, embryoid body; EGF, epidermal growth factor; GFAP, glial fibrillary acidic protein; GFP, green fluorescent protein; hESC, human embryonic stem cell; MBP, myelin basic protein; MP, motoneuron progenitor; OPC, oligodendrocyte progenitors; RA, retinoic acid; RIP, receptor interacting protein; SHH, sonic hedgehog.

Cells were differentiated toward OPC according to already published protocols [10,19] (Fig. 1). Briefly, cell clumps were placed for 2 days in 50% hESC growth media and 50% glial restriction media (GRM) [10] in ultralow attachment six-well plates (Corning, NY, USA, http://www.corning.com). This medium was then replaced with 100% GRM supplemented with 20 ng/ml epidermal growth factor (EGF; Sigma-Aldrich) and 10 μM/ml *all-trans*-retinoic acid (RA) in dimethyl sulfoxide (DMSO) for an additional 7 days. During 25 days the cells were exposed to GRM supplemented with 20 ng/ml EGF. Then, floating yellow spheres were plated in six-well plates (BD, Franklin Lakes, NJ, USA, http://www.bd.com) 1:30 Matrigel for 1 week. The progenitors were migrated from spheres. Cell cultures were then replated on 1:30 Matrigel substrate and cultured for 1 week in GRM supplemented with 20 ng/ml EGF. The duration of the protocol was 42 days. At this point the cells were ready for transplantation. For transplantation, the cells were disaggregated mechanically using a glass pipette.

For motoneuron differentiation, we used modified protocol of Li et al. [12]. Briefly, the H9 cell line was permanently transfected with plasmid carrying GFP (H9-GFP). Cell clumps were placed in normal hESC medium for 4 days without bFGF to form embryoid bodies (EB) in ultralow attachment six-well plates (Corning). The medium was changed daily. Then the EB were placed in normal six-well plates on 1:30 Matrigel substrate to attach in motor induction medium (MIM) composed of DMEM:F12 with Glutamax, N2 supplement, heparin (2 μg/mL), and bFGF (20 ng/mL). After 1 day, EB attached and after 4 days rosettes start to appear. From day 8, 0.1 μM *all-trans*-RA was applied in MIM. The medium with RA was changed daily. At day 16, the rosettes were mechanically cut and placed in ultralow attachment six-well plates as round structure-neuromasses in motor medium (MM) consisting of neurobasal medium, N2 supplement, 1 μM cAMP, 0.1 μM RA, and supplemented with 200 ng/mL sonic hedgehog (SHH; R&D Systems Minneapolis, MN, USA, http://www.RnDSystems.com) for 7 days. The neuromasses were maintained in MM medium supplemented with SHH (50 ng/ml) until the day of transplantation. At the day of transplantation, these neuromass-like structures were disaggregated mechanically with a glass pipette. This is the stage when the early MPs (ISL1+ and Tuj1+) start to mature [12].

### Experimental Groups

To determine whether OPC and/or eGFP-expressing MP are capable to improve motor function when immediately transplanted after SCI, ∼1.5 million cells were transplanted into the spinal cord in the acute phase after a complete transection of the spinal cord at the thoracic level [20,21]. Fourteen rats per group were used. Three different transplantation experiments were performed: the rats were treated with a single cell type (OPC; *n* = 14 or MP; *n* = 14) or in combination (OPC + MP; *n* = 14) and each was followed after transplantation for immunohistochemical evidence of cell incorporation in the lesion site and their survival. We performed electrophysiological and behavioral studies of functional recovery from hindlimb paralysis. We, therefore, defined five groups of animals, including sham and controls (*n* = 14). Acute transplantation controls included animals that received vehicle-only injections. For more details of surgery procedure and other methods used in this study please see Supporting Information.

### Behavioral Assessment

Functional recovery was assessed by evaluators blinded to treatment groups. Open field locomotor test using the Basso-Beattie-Bresnahan (BBB) locomotor rating scale [22] was performed in a plastic tray (50 × 80 × 40). One week before injury, each animal was acclimated to the open field and scored. The BBB test was performed every week after injury during 4 months when two independent examiners observed and recorded, with video digital camera (Sony), the hindlimb movement of the rats, which range from 0 (no hind movement) to 21 (normal gait). The videos were analyzed frame by frame using ImageMixer 3SE software and scored independently by two observers blinded to the treatment group.

### Electrophysiology Measurements In Vivo

The motor potentials were evoked and recorded according to a prior study [23]. The main difference in our study was that the cranial screw was not implanted and a needle electrode was used. According to the anesthetics study of Oria et al. [24], the protocol was administered intravenously as a bolus dose of 10 mg/kg. For the recording of evoked potential (motor-evoked potential [MEP] and compound motor action potential [CMAP]) one needle electrode was placed in the tibialis anterior muscle (cathode) and one-needle electrode subcutaneously at the foot pad level (anode). For the induction of CMAP following peripheral nerve stimulation, one electrode was placed in the muscle (cathode) and another subcutaneously (anode), both near the sciatic nerve. For the induction of MEP (after central stimulation), one-needle electrode was placed subcutaneously at the level of the lower jaw (anode) and a needle electrode (cranial level) was used for the cathode. For ground, an electrode was placed subcutaneously in the lumbar region. The electrophysiological recordings were performed with an electromyographer (Medtronic Keypoint Portable, Denmark) and the bandpass used was 2 Hz to 10 KHz. Throughout the experiments, the duration of the pulse was 0.1 ms. The recordings were started by measuring the maximum amplitude of the CMAP. This was achieved by stimulating the sciatic nerve with a single pulse of supramaximal intensity. To induce MEP, a stimulation of 25 mA intensity was applied at the needle electrode (cranial level). Results were expressed as latency (ms) and amplitude (%) (MEP/CMAP ratio).

### Statistical Methods

BBB scores were analyzed by repeated measures two-way ANOVA with Bonferroni multiple comparison test at each time point. The differences were significant when *p* < .05.

## RESULTS

The experimental procedure including differentiation and cell transplantation is presented in Figure 1. Characterization of hESC-OPC and hESC-MP used for cell transplantation is presented in Supporting Information (Supporting Information Figs. 1 and 2).

### Animals

The majority (80%) of animals survived following injury. Some animals died due to ulcers, autophagia, or considerable weight loss 1 month after surgery. There was a loss of about 10%–20% in animal body weight (data not shown) during the first month, but the animals recovered following 4 months postinjury. No formation of teratoma was observed 120 days after cell transplantation.Complete spinal cord transection lesion was characterized by an obvious traverse scar at the T8 lesion epicenters and neuronal necrosis and cavitations rostral and caudal (below and above) the lesion site (Supporting Information Fig. 3A–E). The transaction site was characterized by the presence of the white tissue between cord stumps. Reactive gliosis was detected by immunohistochemistry using anti-GFAP. These results confirmed that as a consequence of the transection of spinal cord, abundant loss of oligodendrocytes [25] occurred at considerable distance from the lesion.

### Transplanted Cells Survived, Migrated, and Differentiated Within the Spinal Cord

**Figure 2:**
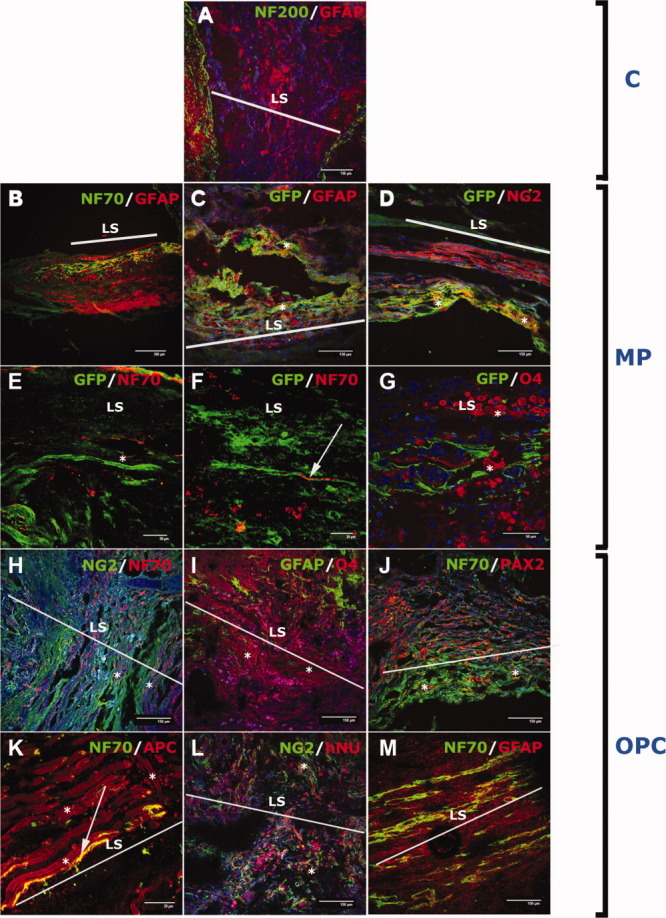
Acute transplantation of human embryonic stem cell-derived OPC or MP resulted in differentiation toward oligodendrocytes and neurons. (**A**): The neuronal fibers (NF200^+^) were stopped at host-scar interface in control (nontransplanted) animals. (**B**): Neuronal fibers positive for human-specific NF70^+^ (green) were detected in the lesion site surrounded with glial GFAP^+^ cells in MP-transplanted animals. In the lesion of the GFP-MP treated animals many GFP^+^ cells were double-labeled with glial GFAP (**C**) and oligodendrocyte (NG2) markers ([**D**], marked as “*”). Dual fates of the transplanted MP were confirmed with double labeling of the GFP (marked as “*”) and NF70 (red; arrow; [**E**] and [**F**]) surrounded by oligodendrocytes (**G**) (marked as “*”). In the lesion site of the OPC-transplanted animals, human-specific neurons were observed because the cells were positive for NF70 ([**H**]; marked as “*”). The majority of the cells in the lesion site were astrocytes, GFAP^+^ (green), and oligodendrocytes O4^+^ (red; [**I**]; marked as “*”). (**J**): Many cells were positive for the human-specific neuronal marker NF70 and interneuronal marker PAX2 (marked as “*”). (**K**): In the lesion site, we observed myelination of transplanted cells. The NF70^+^ fibers (marked as arrow) were surrounded with oligodendrocytes (APC^+^, marked as “*”). (**L**): The majority of the cells were oligodendrocytes and double-labeled with NG2 and hNU (marked as “*”). (**M**): Many NF^+^ fibers were localized only in the lesion site with GFAP^+^ cells. Observations: (**B**): Immunoflourescence was done with the spinal cord samples, where MP (without GFP) were injected. Abbreviations: APC, adematous polyposis coli; GFAP, glial fibrillary acidic protein; GFP, green fluorescent protein; LS, lesion site; MP, motoneuron progenitors; OPC, oligodendrocyte progenitors.

A total of 1.5 million cells were transplanted rostrally and caudally to the lesion site at T8 in 5- to 7-week-old female rats in the acute phase of SCI. We specifically tested three transplantation strategies including control and sham groups. The first group included rats transplanted with GFP-expressed MP (MP group). In the second group of animals, OPCs previously labeled with Hoechst were transplanted (OPC group). In the third group of rats, equal quantity of OPCs was given, previously labeled with Hoechst and GFP-expressing MP (OPC + MP group). In all animals, including the control animals, we administered subcutaneously the phosphodiesterase type 4 inhibitor (Rolipram), an axonal growth supporter [15]. Four months after lesion, control animals treated with or without Rolipram showed only scattered neurofilament (NF)-positive fibers in the central scar. Many NF-immunoreactive fibers were stopped at the host-scar interface (Fig. 2A). Rolipram did not dramatically increase amounts of NF-positive fibers in control rats compared with the rats without Rolipram, in accordance with behavioral tests (see below). The survival of the transplanted cell was less than 1% (Supporting Information Fig. 4). The lesion site of control rats without transplants was negative for anti-human nuclei staining, GFP fluorescence and NF70 (data not shown).

### MP Group

Either GFP-expressing MP or MP, previously dissociated, were injected into the rostral and caudal areas 1–2 mm away from the center of injury to avoid central cavitation. Surviving mature MP-derived GFP cells were observed within the gray matter of the spinal cord (Fig. 2C–2G). Four months after transplantation, GFP-labeled MPs were visualized as a dense mass of elongated, brightly fluorescent cells extending from the lesion site (Fig. 2C). These MP-derived neurons persistently expressed GFP, which confirmed that MPs were capable of surviving and engrafting for at least 4 months after transplantation (Supporting Information Fig. 4). Most of the cells in the lesion were immunoreactive for glial fibrillary acidic protein (GFAP) (Fig. 2B, 2C; 46% ± 6% of GFP^+^). The MPs were gathered in the lesion gap, which was surrounded by an intensively GFAP-positive border of reactive astrocytes reaching 4 mm caudal and 5 mm cranial. GFP and NF70 immunostaining (the lesion samples injected with MP) suggests that these cells differentiated toward neurons in the glial scar (Fig. 2E, 2F; 11% ± 2% of GFP^+^ cells). The phenotype of these neurons remains unknown as these GFP neurons were neither interneurons (Pax2^−^ neurons) nor motoneurons (HB9^−^) 4 months after transplantation (data not shown). Many GFP^+^ cells coexpressed oligodendrocyte markers O4 or NG2 (Fig. 2D, 2G; 36% ± 8% of GFP^+^ cells). An additional proof of dual terminal differentiation of hESC-derived MP is the presence of GFP^+^ cells surrounding NF70^+^ filaments (Fig. 2F) that suggests that transplanted cells also have the capacity to mature to oligodendrocytes.

### OPC Group

Transplanted OPC previously labeled with Hoechst survived and migrated over short distances of the spinal cord during the postimplantation survival period in all treated animals (Fig. 2). Most of the human nuclei positive cells were located in the lesion site (Fig. 2I; Supporting Information Fig. 4). Double staining to human nuclei and GFAP or O4 revealed that the majority of transplanted cells differentiated to astrocytes and oligodendrocytes in the lesion site (Fig. 2I, 2L; 28% ± 3%, 45% ± 6%, respectively). Interestingly, immunohistochemical analysis revealed NF70^+^ fibers in the lesion site suggesting that human OPC also differentiated toward neuronal cells (Fig. 1H, 1J, 1M; 23% ± 4%) often in close association with NG2^+^ cells (Fig. 2H). Specificity of NF70 antibody to human neurons confirms human origin of these cells. Curiously, many adematous polyposis coli (APC)^+^ cells were observed providing myelin sheet to those NF70^+^ fibers (Fig. 2K) suggesting that a considerable number of OPC-transplanted cells differentiated toward neurons and oligodendrocytes (Fig. 1). The NF70^+^ fibers were localized only in the lesion site and ∼2 mm rostrally and 3 mm caudally, suggesting that observed neuronal cells were not motoneurons (Fig. 2A; Supporting Information Fig. 4). Many NF70^+^ cells colocalized with PAX2 (Fig. 2J) suggesting the interneuron nature of differentiated neurons. These observations corroborate with reverse transcription polymerase chain reaction (RT-PCR) analysis of OPC used in transplantation, where we revealed expression of NF and βIII-tubulin (TUJ1^+^) characteristic for neuronal progenitors (Supporting Information Fig. 5).

### OPC + MP Group

**Figure 3:**
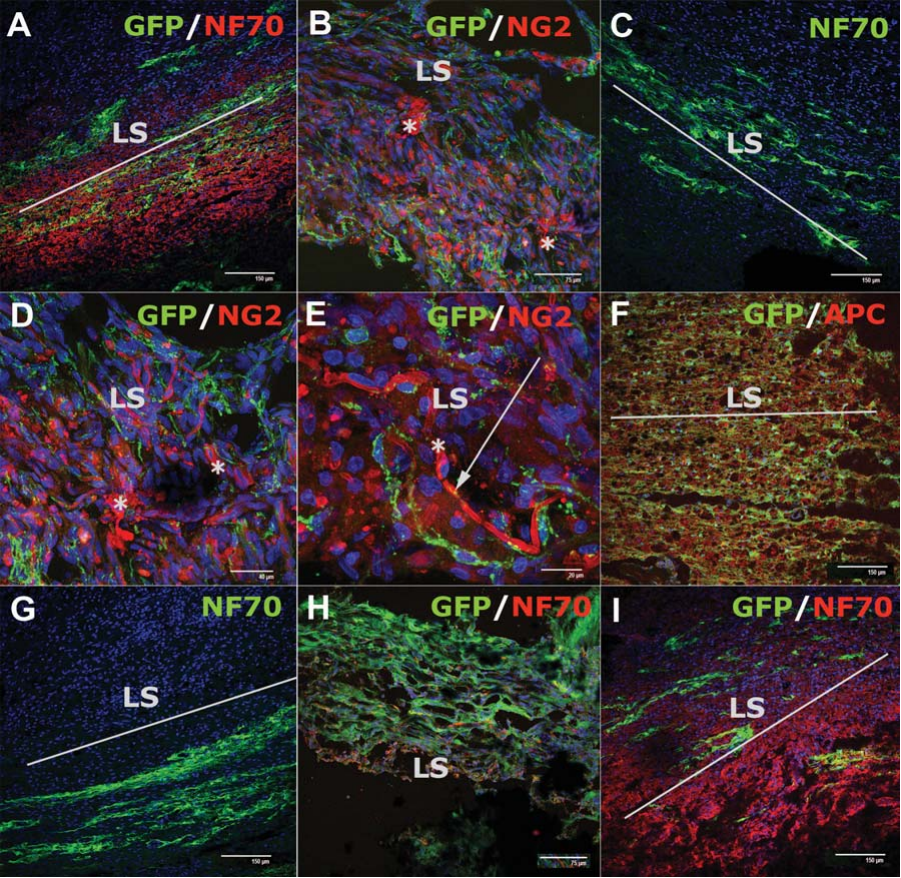
Acute transplantation of human embryonic stem cell-derived oligodendrocyte progenitors (OPC) and motoneuron progenitors (MP) resulted in differentiation toward oligodendrocytes and neurons. Many GFP^+^ cells were observed in the lesion site and colabeled with oligodendrocyte markers NG2 (**A, B, C**) and APC (**D**) (marked as “*”). We observed NG2^+^ (marked as “*”) cells surrounding GFP^+^ (arrow; [**E**]). Many NF70^+^ cells were observed in the lesion site often localized in bundles (**G, H**). All NF70^+^ cells were not GFP^+^ revealing that other human neurons proceed from OPC (**E, F, I**). Observations: (**C, G**): Immunoflourescence was done with the spinal cord samples, where MPs (without GFP) were injected. Abbreviations: APC, adematous polyposis coli; GFP, green fluorescent protein; LS, lesion site.

For the OPC + MP group, we obtained the same immunohistological results regarding the OPC cells and MP. Transplanted cells differentiated to mature oligodendrocytes as confirmed by staining of spinal cord sections for the oligodendroglial markers. Clusters of GFP^+^ cells were double-labeled with both NG2 (Fig. 3A–3C) and APC (Fig. 3D). Detection of APC-CC1-positive cells negative for antihuman nuclear antigen reveals the possible endogenous origin of these cells.

The GFP-positive axons, double-labeled with NF70, were found in OPC + MP transplanted rats (Fig. 2E–2I). NF-positive axons were often found in bundles (Fig. 3E–3G, 3I) and exceeded the number of GFP^+^ cells, thus confirming their OPC origin.

### RT-PCR of the Spinal Tissue

To ensure that the functional recovery was caused by transplanted human cells we isolated total RNA from the lesion site and converted this to cDNA 4 months after transplantation. We found the expression of human *GAPDH*, *GFAP*, *NG2*, *TUJ1*, and *MAP2* but not in the control rats or nonlesioned part of the spinal cord (Supporting Information Fig. 5) confirming the presence of differentiated hESC. These cells finally matured to astrocytes, oligodendrocytes, and neurons (expressing *GFAP*, *NG2*, and *TUJ1*, respectively; Supporting Information Fig. 5) but only OPC and OPC + MP expressed human *MAP2* (marker for postmitotic neurons), which is an additional proof of a triple fate of OPC and MP.

### Behavioral Assessment

**Figure 4:**
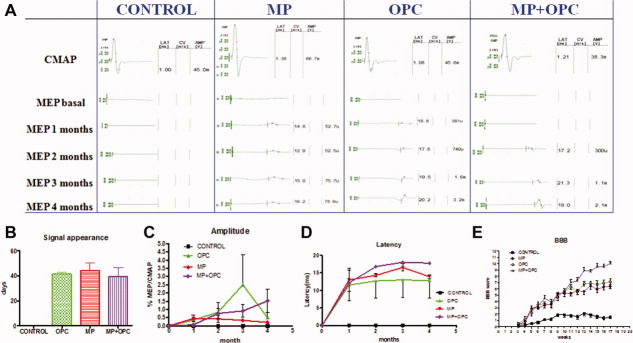
In vivo electrophysiology and BBB test. (**A**): Representative electrophysiological recordings in control and transplanted animals during 4 months of the experiments. (**B**): After ∼40 days, the electrophysiological signal appeared in the transplanted but not in the control animals. Plot graphics of the evolution of amplitude (**C**) and latency (**D**). (**E**): Starting 4 weeks after the transplantation, a significant increase (*p* < .001) in locomotor recovery as determined by the BBB locomotor rating scale was observed in all transplanted animals compared with controls. The values are presented as mean ± SEM. The statistic analysis of BBB score for each time point is presented in Supporting Information Table 1. Abbreviations: BBB, Basso-Beattie-Bresnahan locomotor rating scale; CMAP, compound motor action potential; MEP, motor-evoked potentials; MP, motoneuron progenitors; OPC, oligodendrocyte progenitors.

Hindlimb motor function was assessed using the BBB locomotor rating scale [22,26]. The behavioral results (Fig. 4E) were collected by weekly BBB tests during the 4-month monitoring period for each group of animals. Before the injury, all animals showed normal locomotor activity, scored as 21 on the BBB scale, although all injured rats manifested complete hindlimb paralysis 7 days after injury, resulting in a score of 0. The BBB scores were in the range of 0–1 or 2 in the control animals during the 4 months after SCI. In contrast, MP, OPC, and OPC + MP groups showed hindlimb functional locomotor recovery that increased gradually after 3 weeks of transplantation. Four months after transplantation all 42 transplanted animals displayed BBB scores significantly (*p* < .001) higher than that achieved by the control group. OPC animals reached a final average BBB score of 6, which is significantly (*p* < .001) higher than the control group that reached only 1.5. MP rats partially recovered hindlimb movements and after 4 months reached a final average score of 6, significantly higher than control animals. Also, the OPC + MP group regained significantly (*p* < .001) more functional recovery than the control group and versus single cell type treatment (Fig. 4E) reaching a final average BBB score of 9. Significantly higher (*p* < .001) BBB score in OPC + MP score was reached after 5 weeks of transplantation compared with control animals and after 12 weeks compared with single cell treatment (Fig. 4E and Supporting Information Table 1).

### Electrophysiological Evaluation

Electrophysiological tests were performed in all rats before and after the surgery and transplantation to ensure the complete transection of the spinal cord. MEPs were used to evaluate the function of spinal cord descending tracts [27]. Electrophysiological tests were performed for 4 months in all animals at monthly intervals. The electrophysiological tests demonstrated complete interruption of spinal motor pathways after the injury (Fig. 4A). In control animals, MEP did not recover over time, indicating that interruption of supraspinal axons was maintained until the end of the observation period (Fig. 4A). In contrast, in the OPC group and/or MP group, MEP reappeared ∼40 days after grafting (Fig. 4A, 4B), indicating functional spinal reconnection and partial restitution of motor pathways. Four months after injury, the amplitude of the evoked action potentials showed recovery in OPC and/or MP groups.Comparatively, animals that received OPC and MP tended to show MEP of higher amplitude than those with a single cell progenitor type treatment (Fig. 4C). Although the rats treated with OPC showed higher amplitude after 3 months compared with other treatments, the mean amplitude of evoked potentials after 4 months were higher, but not statistically significant (*p* > .05) in the OPC + MP group compared with OPC or MP (Fig. 4C) groups. Also the animals transplanted with a single type of cells, OPC or MP, showed shorter latency in comparison with OPC + MP (Fig. 4D).

## DISCUSSION

Our results demonstrate that acute transplantation of a single cell progenitor type (OPC or MP) or a combination of thereof into completely transected spinal cord promotes partial recovery of hindlimb movement. As implantation of OPC in the contusion model has been reported to promote partial functional recovery following SCI [10], we focused on upgrading this study to assess the regenerative properties of OPC and MP derived from hESC using an animal model with complete spinal cord transection. To our knowledge, this is the first study that describes cell transplantation of hESC-MP followed by a detailed in vivo electrophysiological assay.

The first signs of recovery in locomotor function were observed after 3 weeks. The BBB scale showed significant improvement of locomotor ability when differentiated hESC were transplanted. The behavior of control animals was consistent with the results obtained using human umbilical mesenchymal stem cells [28] or olfactory ensheathing cells [20]. Because of its non-neurogenic nature, the spinal cord has a nonpermissive environment for the generation of new neurons [29–31] and has a limited capacity for nerve regeneration, at least in part, due to the presence of myelin-associated inhibitors that trigger signaling pathways that decrease intraneuronal cAMP [32]. To stimulate axon regeneration, the inhibitory effects of myelin need to be compensated by dibutyryl cyclic AMP (dbcAMP) and systemic administration of Rolipram [15,16,33]. The effectiveness of this phosphodiestherase inhibitor after SCI is already known [15,34,35] and it could attribute additionally to the neuronal growth of transplanted cells and locomotor recovery of lesioned rats after cell transplantation. Passive and active daily rehabilitation of injured animals could favor the recovery. Although the mechanisms by which the training could improve the performance in lesioned rats are still unknown, training could facilitate axon regeneration, and/or provide protection for the neurons and glia near the injury site increasing neurotrophic factors [brain-derived neurotrophic factor (BDNF), NT-3] and their Trk receptors in the spinal cord [36–39].

A novelty of this study is that after spinal cord transection OPC- and/or MP-transplanted rats showed recovery of MEP. Immediately after complete transaction, MEPs disappear because of disruption of all descending tracts. In the control group, we did not observe MEP throughout the experiment due to the failure of spontaneous axonal regeneration within nontransplanted spinal cord [40]. The recoveries of MEP can be attributed to the reconnection of the axons above and below the lesion site and the contribution of oligodendrocytes. The time of reappearance of MEP in treated animals is associated with the stabile partial recovery observed by BBB, suggesting that recovery of motor skills was due to the recovery of descending control of hindlimb movements.

We determined that MP are able to mature and develop fundamental functions of normal motoneurons in vitro (expressing *OLIG2, ISL1*, and *HOXC5*), including directional growth of long axons, confirming the results from the previous study [11]. The BBB score of the MP group was higher than in the control group and immunohistochemistry analysis confirmed that hESC-MP survive, migrate, and engraft for at least 120 days in the lesion site. These data suggest that the application of ex vivo conditioning may allow efficient generation of new neurons in non-neurogenic regions as it is SCI. Interestingly, the immunohistochemistry analysis showed clear evidence that these progenitors have the capacity to finally differentiate to mature oligodendrocytes and neurons in the lesion site. Our strategy did not result in the formation of anatomically, physiologically, and functionally active motor units between transplanted axons and host muscle, but the fact that these cells innervate the lesion site filling the gap between the rostral and caudal stumps as well as significantly improved locomotor function of lesioned rats suggests that hESC-MP have promising regenerative potential.

Interestingly, transplanted OPC had significant recovering effects too. In a contusion model, even after severe contusive SCI, surviving axons persist in the subpial rim of white matter and restoration of the oligodendrocyte population by replacement therapy has been considered as a potentially attractive strategy to promote remyelination after SCI [10,19,41]. But in the case of the spinal transection model there is no evidence of the presence of spared host axons. After analysis of control rats, survival of host axons or spontaneous regeneration was not observed. Immunohistochemical analysis showed differentiation of transplanted cells toward neuronal cells, which was confirmed by the presence of human specific NF70^+^ neurofilaments in the lesion site. This result contrasts to previous claims that hESC differentiate exclusively toward a pure population of OPC [10,19]. We hypothesized that the potential of OPC to recover is due to the presence of heterogeneous cell types or multiple character of transplanted progenitors. Analyzing the transcription phenotype of obtained OPC at day 42, we observed strong expression of *OLIG2* [42–44], which gives rise to motoneurons during the neurogenic phase. Nevertheless, OLIG2 is expressed in human OPC [45] and is necessary for specification and differentiation of human OPC [44]. In rodents, OLIG2-expressing spinal progenitors from the MP (pMN) domain are a source of both motoneurons and OPC [46,47]. pMN cells in the spinal cord initially produce motoneurons, but switch later in development to form oligodendrocytes [48–50]. It has been proposed that transplanted OPC may undergo their last division to adapt to a new environment by responding appropriately to environmental cues [51] (Fig. 1). Shihabuddin et al. [29] have showed that isolated and in vitro expanded neural progenitors from adult spinal cord (non-neurogenic zone) have the ability to respond to epigenetic signals in vivo, after transplantation, by site-specific differentiation, generating proper neurons and/or glial cells depending on the transplantation site. Although we can conclude the commitment of generated OPC to oligodendroglial fate [10,19], neuronal cells were also observed in vitro indicating that nearly 20% of differentiated cells were TUJ1^+^. We believe that the presence of neuronal progenitors within transplanted OPC caused significant recovery of animals. OPC could generate a paracrine/trophic environment and positively modulate the local immune response, as well as promote neuronal protection and activation of endogenous neurogenesis [41,52] suggesting that regenerative mechanisms do not depend exclusively on a specific cell fate. Possible percentage of Tuj+ (spontaneously generated or not) could have origin from early neural processes. By the way, all cells arise from the same pool of neural stem cells, and it is not excluded that neurons arose from this pool. Finally, the cell transplantation strategy showed similar recovery effects that included synergic action of OPC and MP because these two cells types formed myelin-axon interaction.

The functional locomotor recovery analysis of the rats transplanted with OPC and MP showed significantly better hindlimb recovery than the animal groups treated with a single cell type. These results have shown that combination of OPC and MP is superior approach when comparing with a single type strategy used in our study or when compared with the studies where other cell stem types strategies used in the same rat model of SCI [20,28,53,54]. As demonstrated in our results, locomotor improvement after transplantation, the OPC and MP is associated with abundant presence of human NF-positive fibers and oligodendrocytes in the lesion. That means that hESC-derived OPC and MP were able to differentiate into mature oligodendrocytes and neurons. This was not the case in our previous study where we used ependymal stem cells to treat animals with spinal cord contusion [41].

## CONCLUSION AND PERSPECTIVES

Therapeutic use of stem cells to regenerate neural cells in the damaged spinal cord requires efficient delivery of stem cells into the injured area. The cells should differentiate into mature and functional cells that engraft for extended periods of time. Future studies must examine: (a) whether hESC-derived motoneurons have the ability to extend toward anatomically and functionally appropriate distal muscle targets; (b) whether they can form junctions on reaching these muscles as previously shown using mESC [15]; and (c) whether the functional locomotor improvement can be enhanced using OPC. Therefore, the mechanisms underlying the promotive effects on the functional recovery after transplantation of the hESC-derived OPC and MP is rather the ability of these cells to differentiate into neuronal and glial cells than promotion of endogenous axonal regeneration.

Taken together, our study demonstrates that hESC-OPC and hESC-MP, when transplanted into the spinal cord immediately after the injury, survived for at least 4 months; migrated at least 3 mm away from the lesion; differentiated into appropriate cell types without forming teratomas, and improved locomotor function. These results open up new possibilities and strategies for future clinical applications in SCI and constitute a promising approach for repairing the damaged spinal cord especially if transplantation is shortly delayed after the lesion.

## Disclosure of Potential Conflicts of Interest

The authors indicate no potential conflicts of interest.
