# Supplementary figures and images for: Transplanted Oligodendrocytes and Motoneuron Progenitors Generated from Human Embryonic Stem Cells Promote Locomotor Recovery After Spinal Cord Transection
Source: Stem Cells. 2010 Jul 27;28(9):1541–9. doi: 10.1002/stem.489 (PMC2996083; doi:10.1002/stem.489)

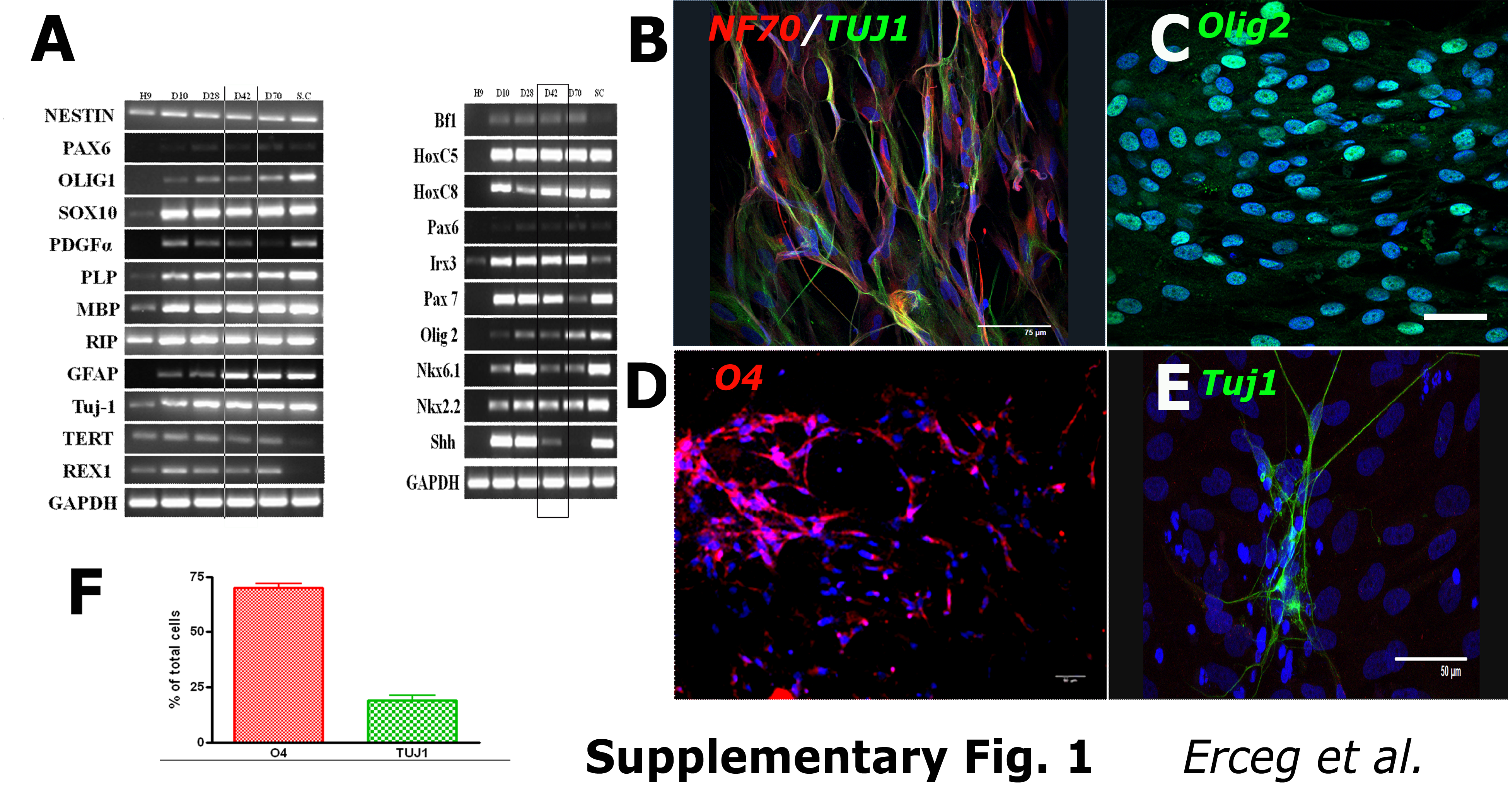

Supplement: Supplementary file 1 [file stem0028-1541-SD1.tif]

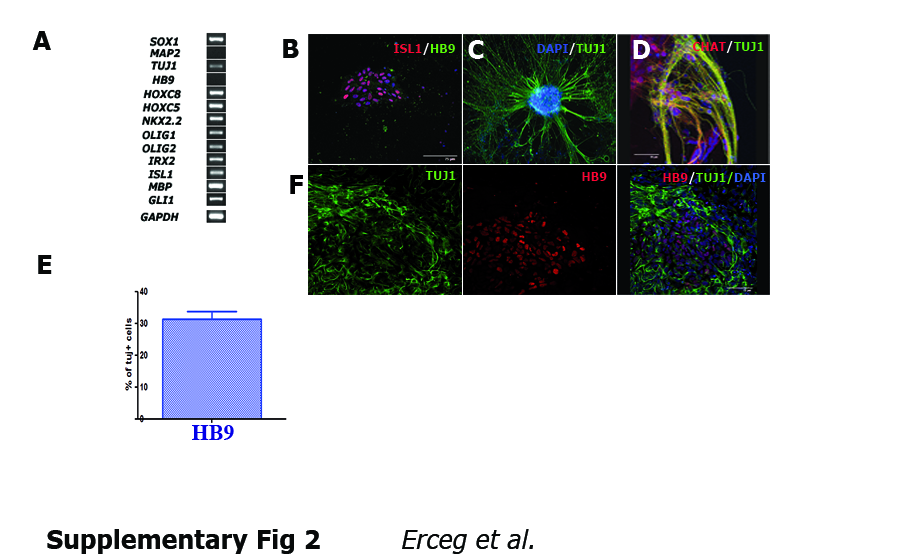

Supplement: Supplementary file 2 [file stem0028-1541-SD2.tif]

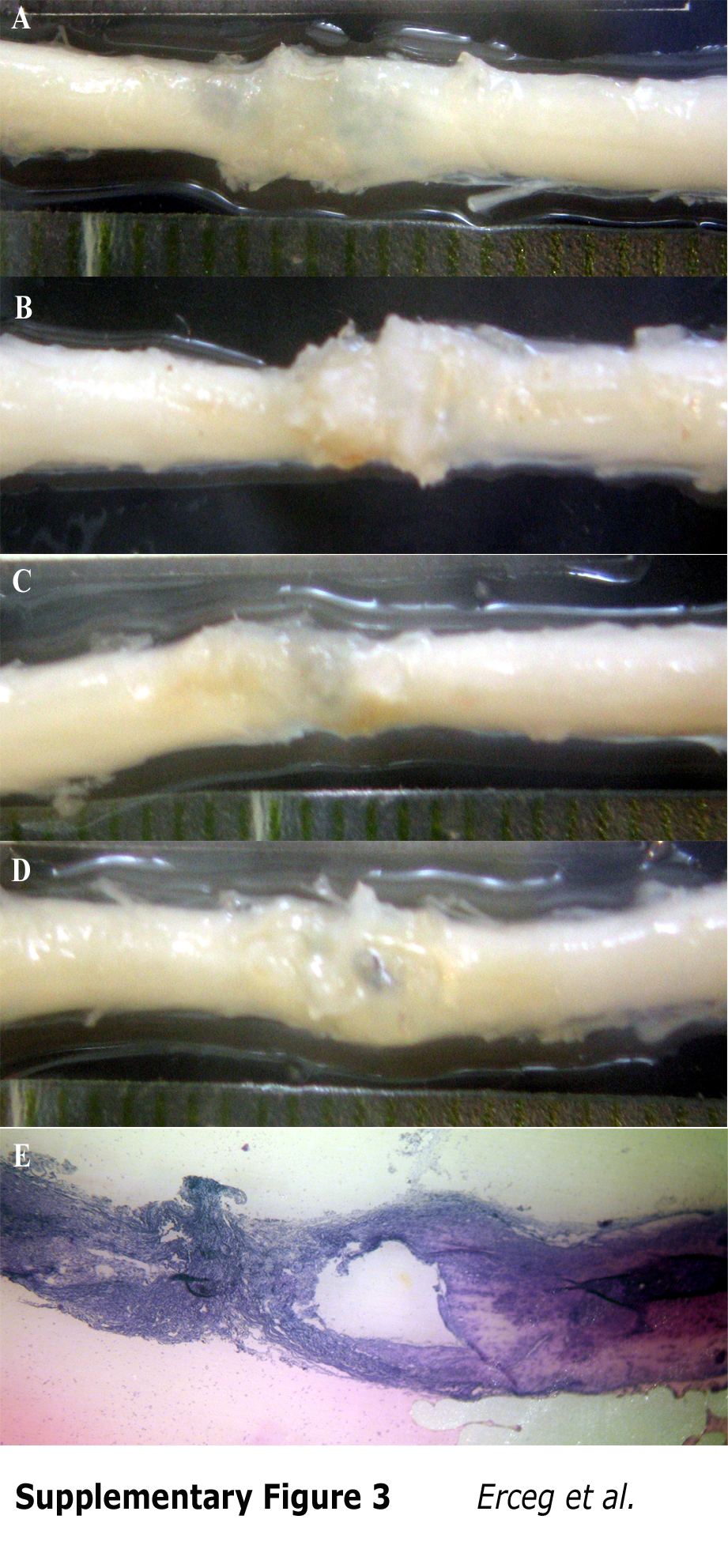

Supplement: Supplementary file 3 [file stem0028-1541-SD3.tif]

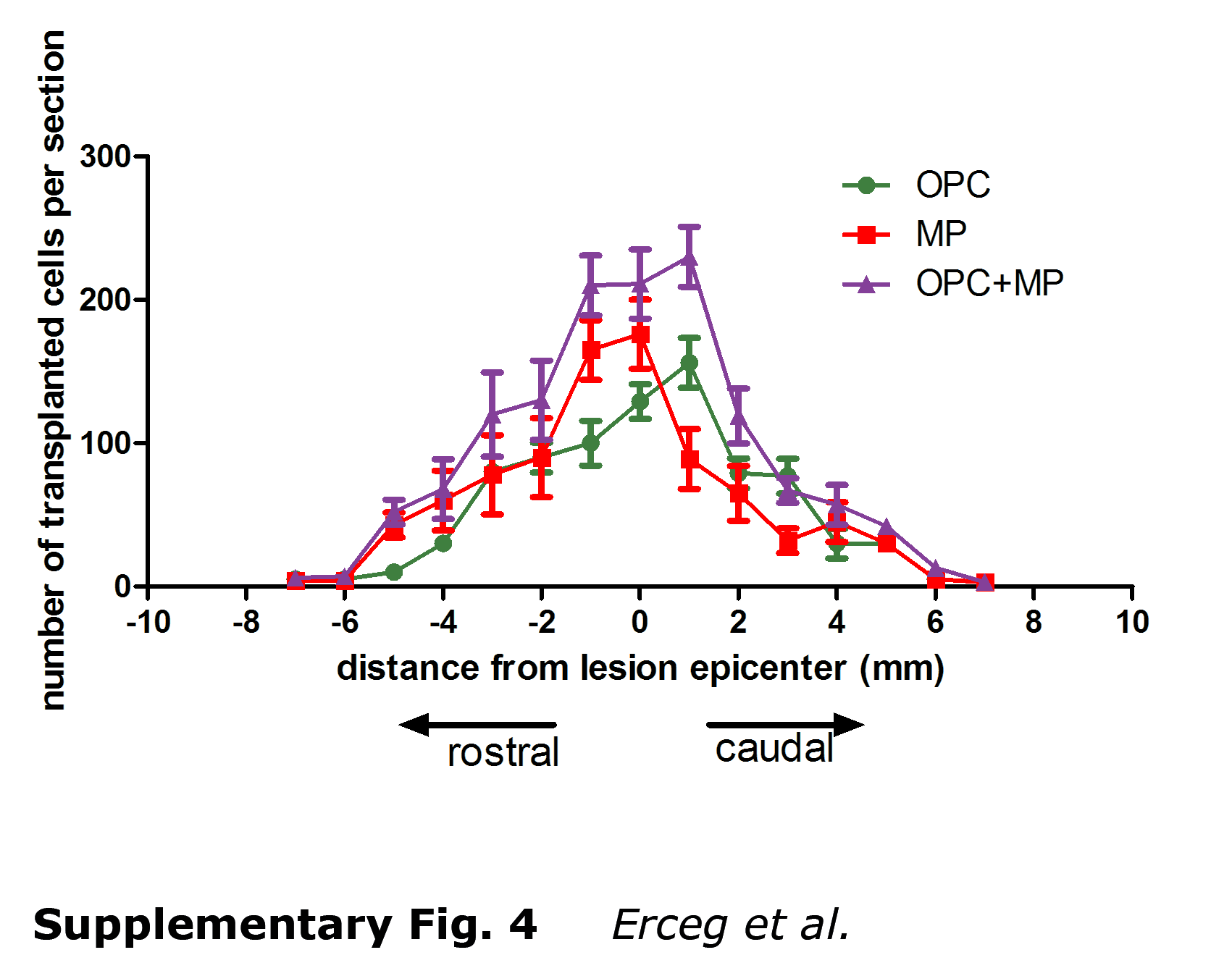

Supplement: Supplementary file 4 [file stem0028-1541-SD4.tif]

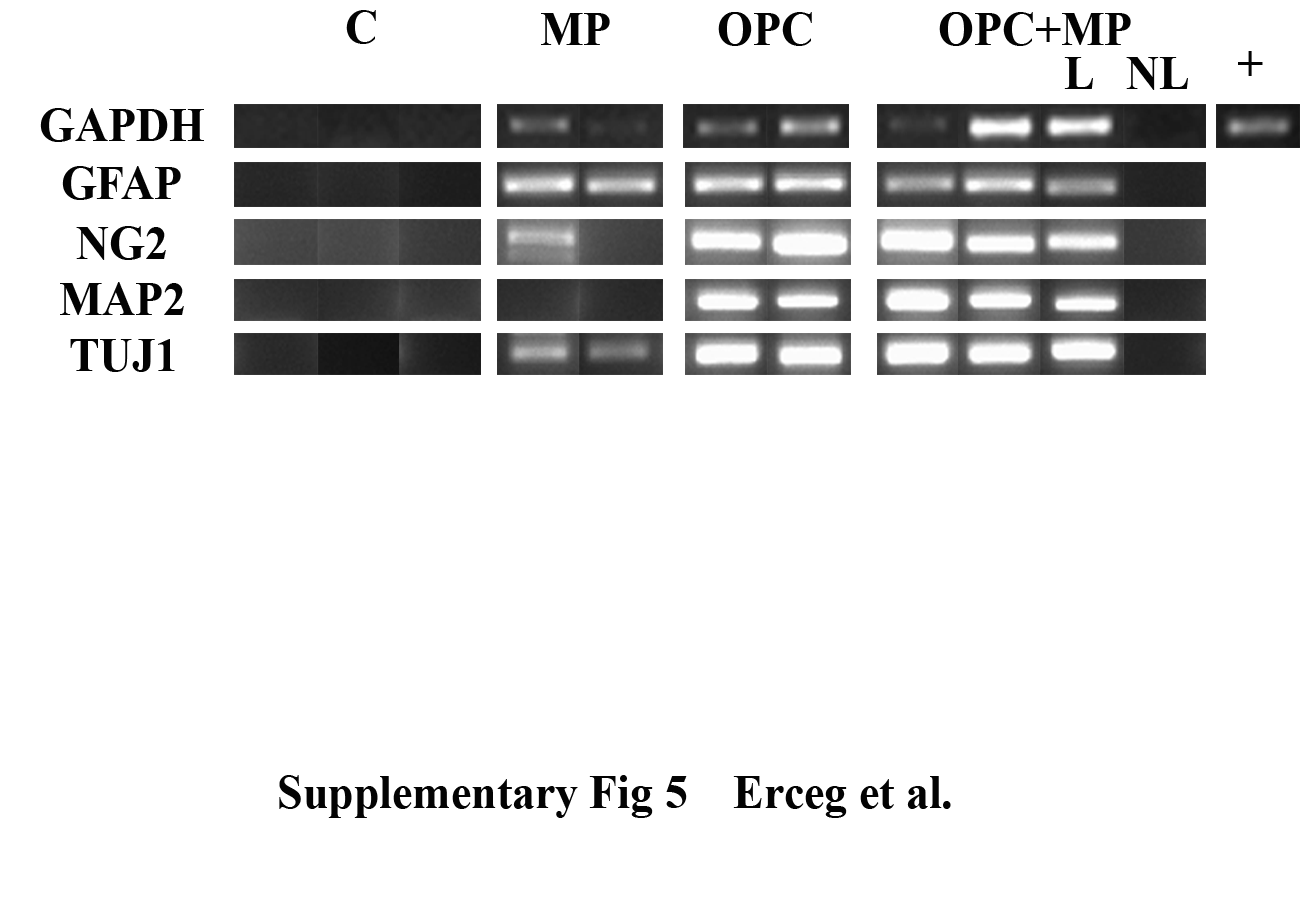

Supplement: Supplementary file 5 [file stem0028-1541-SD5.tif]
