# Supplementary material for: Transplanted Oligodendrocytes and Motoneuron Progenitors Generated from Human Embryonic Stem Cells Promote Locomotor Recovery After Spinal Cord Transection
Source: Stem Cells. 2010 Jul 27;28(9):1541–9. doi: 10.1002/stem.489 (PMC2996083; doi:10.1002/stem.489)
Supplement: Supplementary file 6 [file stem0028-1541-SD6.doc]

| **weeks** | **c vs OPC** | **c vs MOTO** | **c vs. MOTO+OPC** | **MOTO vs. OPC** | **OPC vs. OPC+MOTO** | **MOTO vs. OPC+MOTO** |
| --- | --- | --- | --- | --- | --- | --- |
| 3 | ns | ns | ns | ns | ns | ns |
| 4 | ns | ns | ns | ns | ns | ns |
| 5 | ns | *** | *** | ns | ns | ns |
| 6 | *** | ** | *** | ns | ns | ns |
| 7 | *** | ** | *** | ns | ns | ns |
| 8 | ** | *** | *** | ns | ns | ns |
| 9 | *** | *** | *** | ns | ns | ns |
| 10 | *** | *** | *** | ns | ns | ns |
| 11 | *** | *** | *** | ns | ns | ns |
| 12 | *** | *** | *** | ns | * | * |
| 13 | *** | *** | *** | ns | ** | *** |
| 14 | *** | *** | *** | ns | *** | ** |
| 15 | *** | *** | *** | ns | *** | ** |
| 16 | *** | *** | *** | ns | *** | ** |
| 17 | *** | *** | *** | ns | *** | ** |

## Erceg et al., Supplementary Table 1
