# Supplementary material for: Transplanted Oligodendrocytes and Motoneuron Progenitors Generated from Human Embryonic Stem Cells Promote Locomotor Recovery After Spinal Cord Transection
Source: Stem Cells. 2010 Jul 27;28(9):1541–9. doi: 10.1002/stem.489 (PMC2996083; doi:10.1002/stem.489)
Supplement: Supplementary file 7 [file stem0028-1541-SD7.doc]

***Characterization of hESC-OPC and hESC-MP used for cell transplantation***

After 42 days OPC were generated by applying the differentiation protocol described by Nistor et al.^1^ and Keirstead et al.^2^. Immunofluorescent staining demonstrated that the purity of OPC was between 65% and 70%. RT-PCR analysis showed that the cell population prepared for transplantation expressed i) genes specific for oligodendrocyte progenitors: *SOX10, RIP, PLP, MBP,* and *PDGF* (**Suppl**. **Fig. 1A**), ii) neural lineage *OLIG1, OLIG2* as well as iii) genes characteristic for spinal motoneuron lineage: *HOXC5*, *HOXC8*, *NKX2.2*, *NKX6.1*, *IRX3* (**Suppl. Fig. 1A**). This was confirmed 14 days after plating the cells indicating that the majority of these neural progenitors adopted early oligodendroglial fate as well as early neuronal fate. The cells expressed oligodendrocyte markers **(Suppl. Fig. 1A** and **1D)** but a considerable population of neuronal progenitor were observed too; 19±5% of the total cells were positive for TUJ1 and neurofilament (**Suppl. Fig. 1A, 1B** and **1E**). Also, strong expression of *OLIG2*, the helix-loop-helix transcription factor, normally expressed in neuroectodermal cells in response to ventrally derived sonic hedgehog (SHH) was observed (**Suppl. Fig. 1A** and **1D)**. The OPC also expressed O4 (65±5%) at day 42. The cells also had a bipolar morphology, characteristic of neural progenitors ref (**Suppl. Fig.** **1D**).

After treatment with RA at the early stage (day 8-10), motoneuron progenitors were cultured in the presence of SHH for 7 days. The neuromass-like structures were maintained in motor medium (MM) ^3^ supplemented with SHH for 7 days. At this stage the cells were disaggregated and transplanted in rats with SCI. RT-PCR analysis of the cells used for transplantation revealed spinal motoneurons characteristic of generated progenitors expressing: *ISL1, TUJ1, OLIG1, OLIG2, SOX1*, *HOXC5*, *HOXC8*, *IRX2*, *NKX2.2* (**Suppl. Fig. 2A** and **2D)** but also markers for oligodendrocyte lineage such as MBP **(Suppl. Fig. 2A)**. Only a few ISL1^+^ cells were co-labelled with HB9 (**Suppl. Fig. 2B**), a motoneuron marker, which is in concordance with already published data ^3, 4^ . It is well known that human motoneuron progenitors do not survive *in vitro* as disaggregated cells ^5^. For terminal differentiation neuromass-like structures were plated on poly-ornithine/laminine maintained in MM differentiation medium with the addition of BDNF, GDNF, IGF1 and SHH. Under such growth conditions, HB9^+^ cells were present in culture **(Suppl. Fig. 2E)**. After 3 weeks in final MM (FMM) cells also expressed choline acetyltransferase (ChaT) **(Suppl. Fig. 2D)**. The ChAT^+^ cells were largely localized to the cluster and were βIII-tubulin^+^ neurons **(Suppl. Fig. 2D)**.

**Supplementary Figure legends**

**Supplementary** **Figure 1**

RT-PCR analysis and immunocytological characterization of the generated OPC progenitors used for transplantation. (**A**) RT-PCR analysis of the 70 day protocol of differentiation of oligodendrocytes from hESC, indicated changes in the main genes involved in spinal motoneuron and oligodendrocytes generation. Day 42 of the protocol is in the frame. (**B**) and (**E**) After 42 days of differentiation, OPC immunocytochemistry analysis revealed many TUJ^+^ (green) and NF70^+^ (red) cells. (**C**) OLIG2-expressing progenitors on day 42. (**D**) After 70 days of the protocol on laminin-poly lysine matrix, the majority of OPC progenitors were positive for O4. (**F)** Percentage of Tuj 1^+^ and O4 + cells after 70 days of the protocol. Abbreviation: SC- human spinal cord

#### Supplementary Figure 2

#### RT-PCR analysis and immunocytological characterization of the generated motoneuron progenitors used for transplantation. (A) RT-PCR analysis of the neuromass-like cells prior to transplantation after successive RA and SHH treatment. On day of transplantation the majority of cells express *ISL1* (B, red) and only a few are co-labelled with HB9 (B, green). (C) After 3-4 weeks on laminin/ornithine matrix the majority of cells were TUJ^+^ (C) coexpressed with CHAT^+^ (D) and HB9 (F). (E) Percentage of Tuj1^+^ cells expressing HB9 cells after 3-4 weeks on laminin/ornithine matrix.

#### Supplementary Figure 3

#### Representative macroscopic images of completely transacted spinal cord 4 months after injury. Control group (A), OPC group (B), MP group (C), and OPC+MP group (D). (E) Histological architecture of the lesion site after 4 months presented by Eosin-haematoxilin staining.

#### Supplementary Figure 4

#### Distribution of hNU (OPC, OPC+MP) and GFP (MP) immunostained transplanted cells in transverse sections 4 months after transplantation. Error bars illustrate mean±s.e.m.

#### Supplementary Figure 5

Human specific RT-PCR analysis of spinal cord tissue of control and transplanted animals. In all three control rats (n=3) the presence of human GAPDH was not observed, while in all transplanted animals: OPC (n=2), MP (n=3), MP+OPC (n=3) as well as control positive tissue (human spinal cord, “+”) GAPDH was present. The presence of human GAPDH in the caudal region 4 cm above the lesion site (NL) of the MP+OPC group was not observed. Abbreviation: NL-non lesioned region of MP+OPC group , L- lesion site tissue of the same animal.

#### Supplementary Table 1

Two way Anova statistic analysis locomotor test BBB of the different time point and the comparison of the treatments used in our study: Control vs. cell treatments, MOTO+OPC vs. OPC, and MOTO+OPC vs. MOTO. Abbreviations: * P<0.05, ** P<0.01, *** P<0.001, ns: not significant.

**Supplementary MM**

***Immunocytochemical and immunohistochemical staining***

To determine the gene and protein expression profile of transplanted cells we performed immunocytochemical staining of the cells on the day of transplantation. Also to determine the terminal differentiation of derived cells, OPC were plated on poly-L-lysine- and human laminin- (Sigma-Aldrich) coated slides in GRM medium with EGF while MP were plated on poly-ornithine- human laminin plates in final motoneuron medium (FMM) consisting of MM medium supplemented with BDNF (20 ng/mL), GDNF (20 ng/mL) and IGFI (20 ng/mL). Media was changed every other day. All the cells were concentrated to 100,000 cells/µl. Trypan blue exclusion testing indicated that this preparation consisted of 89-97% viable cells (data not shown).

For immunohistochemistry analysis, animals were killed 4 months after cell transplantation under pentobarbitone anaesthesia and were perfused with 4% paraformaldehyde (Fisher Scientific) in 0.1 M phosphate buffer, pH 7.4, for cryostat processing. Cryostat sections were used to determine the spread of hNU^+^ and GFP^+^ transplanted cells, the distribution of anti-human immunostained cells, the differentiation profile of transplanted cells, and morphometric assessments of tissue sparing/loss.

For immunocytochemistry and immunohistochemistry analysis we used the following antibodies: rabbit anti-OLIG2 (1:100), rabbit anti-OLIG1 (1:200), mouse anti-GFP (1:150), rabbit anti-GFP (1:200), rabbit anti-NF70 (1:150), mouse anti-NF70 (1:250), mouse anti-NF200 (1:250), mouse anti-hNU (1:100), rabbit anti-APC (1:200), rabbit-anti PAX2 (1:200), mouse anti-CHAT (1:150), mouse anti-VAChT (1:150), rabbit anti-NG2 (1:250), (all from Chemicon, Temecula, CA), mouse anti-class III β-tubulin (Tuj1; 1:200, Abcam), rabbit anti-cow GFAP (1:500; DakoCytomation, Glostrup, Denmark), using standard protocols ^6^. The percentage of immuno-positive cells was determined by dividing the total number of immuno-positive cells by the total number of DAPI-positive cells in each imaging chamber and averaging the results from four imaging chambers per marker. To determine distribution of transplanted cells and to perform quantitative cell survival analysis , the human nuclei immunopositive (in the case of OPC and OPC+MP) and GFP positive cells (in the case of MP) were counted on three sections 100 µm apart from each tissue block for each animal in the parasagittal plane and averaged. All GFP cells with the average diameter of the cell body of 6 µM in each section were counted. For assessment of possible differentiation of hESC derived OPC and MP into mature neurons, oligodendrocytes and astrocytes section containing GFP-labelled transplanted cells or hNU positive cells were double stained with NF70, O4 and GFAP and counted. Quantification of the nerve fibres in the transection site of the spinal cord were observed under optical microscope (Zeiss axiovert) and analysed by *ImagePro* software.

***Surgical procedure and cell transplantation***

Female Adult Wistar rats (150-200 gr) were injected with cyclosporin A (10 mg/kg/d, s.c.; Bedford Laboratories, Bedford, OH) beginning 1 day before transplantation until the end of the study. Rolipram (Merck, Calbiochem) was administered at 0.5 mg/kg/day subcutaneously beginning 2 days before surgery and cell transplantation and continuing for 30 days after transplantation.

Before the surgery the animals were pre-medicated subcutaneously with morphine (2.5 mg/kg). Then the rats were placed in an induction chamber to which 4% isofluorane in a continuous oxygen flow of 1 L/min was applied. Once the animals were anaesthetized, they were placed in an anaesthetic mask with isofluorane 1-2% in a continuous flow of 0.2-0.4 L/min of oxygen. The dorsal area between the neck and hindlimbs extending ~2 cm bilaterally from the spine was shaved and disinfected with serial povidone. Under aseptic conditions, a longitudinal dorsal midline skin incision was made over the spinal column from T5-T11 , and the muscles overlying the vertebral column were reflected exposing the vertebral column T7-T10. Then the rats were subjected to a laminectomy at the T8-T9 thoracic spinal cord segments and complete transection of the spinal cord was performed with micro-scissors. Prior to complete transection, the lesion site was treated with Lidocaine. The rostral and caudal stumps were carefully distracted and the cavity was carefully explored with a glass probe to cut any residual fibers and to verify complete transection. It has been reported also that 5-10% of spared white matter in the ventrolateral funiculi is sufficient for sustained rat locomotion ^7, 8^. Immediately before transplant, the cell transplant population was assayed for viability by assessing trypan blue exclusion and only cell populations with >95% viability were transplanted. After immobilization on a spinal cord device (Kopf Instruments, USA), a 10 µl Hamilton syringe (Hamilton, Reno, NV) with a silicon-coated pulled 100 μm glass tip was lowered into the spinal cord using a stereotaxic manipulator arm and injection unit. The rats received 4 injections of the corresponding cells: two injections 1 mm cranial and two injections caudal to the lesion epicentre. Cell suspensions were injected at a rate of 2 µl/min. Animals in which the injected solution was seen to exit from the needle track during injection or after withdrawal of the needle were omitted from the experiment (and are not represented in the experimental numbers). The host spinal cord cranial and caudal to the lesion epicenter was targeted to avoid the epicenter of cavitation, hemorrhagic necrosis, and inflammation, which might decrease cell survival and integration and to target the penumbra of the lesion. The needle was removed after 5 min. Control animals received vehicle only.

The deep and superficial muscle layers were sutured, and the skin was closed with 4/0 sterile suture. Immediately after surgery, animals were given subcutaneous saline and the antibiotics Baytril (2.5 mg/kg/d, s.c.; Bayer, Shawnee Mission, KS) and Buprex (0,1 mg/kg, s.c. Schering-Plough) and maintained on an isothermic pad until alert and mobile. Bladders of all injured rats were expressed manually twice daily until bladder function was restored within weeks post-injury. The rats were inspected for weight loss, dehydration, discomfort, and autophagia, with appropriate veterinary care as needed. The treatment with Baytril was maintained 7 days after surgery to prevent possible infections. The rats whose weight was declined more than 20% were excluded from experiments (and are not represented in the experimental numbers). The studies were approved by the CIPF Animal Research Committee and followed the Animal Care Guidelines.

***Animal care***

After surgery, during 15 days all the animals were subjected to daily rehabilitation procedures consisting of passive mobilization through a full range of movement to maintain joint flexibility, and reflexes in the hindlimbs were assessed i.e., withdrawal reflex, toe spread response and massage of hindlimbs for 15 min every day. After 15 days, in addition to passive mobilization, the daily rehabilitation included active mobilization on a treadmill (Cibertec), with a rolling belt with adjustable speed (up to 150 cm/s) and slope (from 0 to 25 degrees), and swimming in a pool. Animals performed daily quadripedal locomotion on a treadmill until they could maintain a forward position on the belt moving at 11-13 m/min and continuously drink from a liquid dispenser containing sugar water. Negative reinforcement (tail shock) was not used but the device has an electrical shock supplied by the grid.

One week prior to surgery, animals were exposed to the swimming pool and treadmill for several minutes per day for 5 consecutive days. During this time the research personnel responsible for swimming and testing handled the animals on a daily basis and fed them sweetened cereal as treats. Rats are natural swimmers and were quickly acclimatized to the swimming sessions. A swim test was performed in a rectangular Plexiglas basin (70 cm long, 45 cm wide and 25 cm deep) filled with temperate water (35ºC). Intact animals swim by paddling with their hindlimbs and the tail, holding their forelimbs immobile under the chin ^9^.

After each pass, animals were removed from the end of the tank and given a short rest, the length of which depended on their past performance. These procedures facilitated a straight swimming trajectory across the tank and prevented escape behaviours. In addition, rats received intermittent positive reinforcement for successful trials (sugared cereal). Initially after SCI, rats required a vest with narrow strips of closed-cell foam on the back to assist with flotation, and they primarily used the forelimbs to swim. The foam was sufficient to keep the head above the water only when the rats swam. The foam was removed after 4-5 sessions when swimming movements of the hind limbs began to emerge and were sufficient to keep the rat afloat. Daily notation of hindlimb swim performance included extent of movements and relative frequency of kick cycles. Time engaged in swimming was recorded for each animal to measure training effects. Rest periods of up 2 minutes after each bout of swimming were provided early after SCI to avoid inducing marked stress and non-compliance of the rat. Rest times were gradually shortened to 1 min as the hindlimb swimming movements increased.

***RNA Extraction and Reverse Transcription-PCR Analysis***

To extract the RNA from the tissue, the lesion site including 3 mm above and below the lesion was cut from the three rats of all experimental groups and homogenized by Tissueruptor (Qiagen, USA). The total RNA was extracted using RNeasy mini kit (Qiagen, USA) according to the manufacturer's instructions. For cells, total RNA was extracted using High Pure RNA isolation Kit according to the manufacturer's instructions (Roche Diagnostics). cDNA was synthesized using High Capacity cDNA Archive Kit (Applied Biosystems, CA, USA). Amplification was performed on the cDNA using Taq polymerase (Invitrogen). PCR conditions included a first step of 3 min at 94°C, a second step of 15 sec at 94°C for 35 cycles, a 30 sec annealing step at 56°C, 45 sec at 72°C, and a final step of 8 min at 72°C. Glyceraldehyde-3-phosphate dehydrogenase (GAPDH) was used as a control gene to evaluate and compare the quality and quantity of different cDNA transcripts. Primer sequences can be provided upon request.

**Supplementary references**

**1.** Nistor GI, Totoiu MO, Haque N, Carpenter MK, Keirstead HS. Human embryonic stem cells differentiate into oligodendrocytes in high purity and myelinate after spinal cord transplantation. Glia*.* Feb 2005;49(3):385-396.

**2.** Keirstead HS, Nistor G, Bernal G, et al. Human embryonic stem cell-derived oligodendrocyte progenitor cell transplants remyelinate and restore locomotion after spinal cord injury. J Neurosci*.* May 11 2005;25(19):4694-4705.

**3.** Li XJ, Du ZW, Zarnowska ED, et al. Specification of motoneurons from human embryonic stem cells. Nat Biotechnol*.* Feb 2005;23(2):215-221.

**4.** Lee H, Shamy GA, Elkabetz Y, et al. Directed differentiation and transplantation of human embryonic stem cell-derived motoneurons. Stem Cells*.* Aug 2007;25(8):1931-1939.

**5.** Singh Roy N, Nakano T, Xuing L, Kang J, Nedergaard M, Goldman SA. Enhancer-specified GFP-based FACS purification of human spinal motor neurons from embryonic stem cells. Exp Neurol*.* Dec 2005;196(2):224-234.

**6.** Erceg S, Lainez S, Ronaghi M, et al. Differentiation of human embryonic stem cells to regional specific neural precursors in chemically defined medium conditions. PLoS ONE*.* 2008;3(5):e2122.

**7.** Basso DM, Beattie MS, Bresnahan JC. Graded histological and locomotor outcomes after spinal cord contusion using the NYU weight-drop device versus transection. Exp Neurol*.* Jun 1996;139(2):244-256.

**8.** Schucht P, Raineteau O, Schwab ME, Fouad K. Anatomical correlates of locomotor recovery following dorsal and ventral lesions of the rat spinal cord. Exp Neurol*.* Jul 2002;176(1):143-153.

**9.** Liebscher T, Schnell L, Schnell D, et al. Nogo-A antibody improves regeneration and locomotion of spinal cord-injured rats. Ann Neurol*.* Nov 2005;58(5):706-719.
